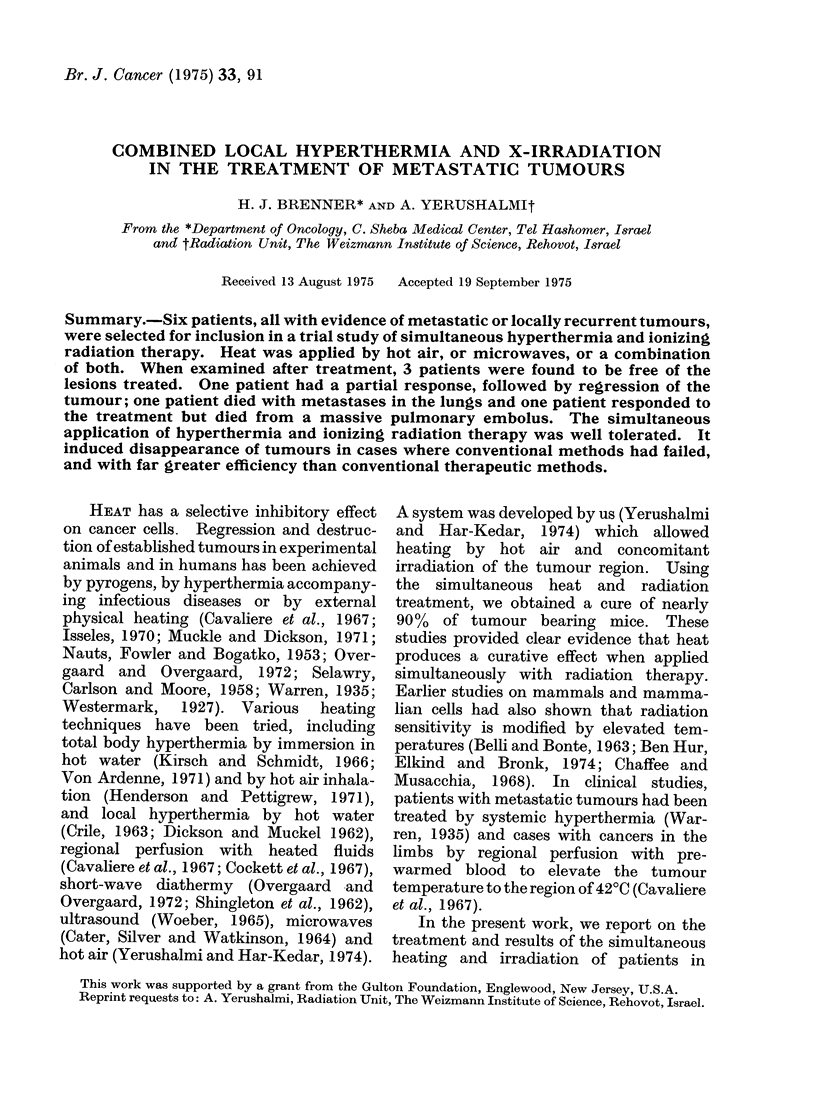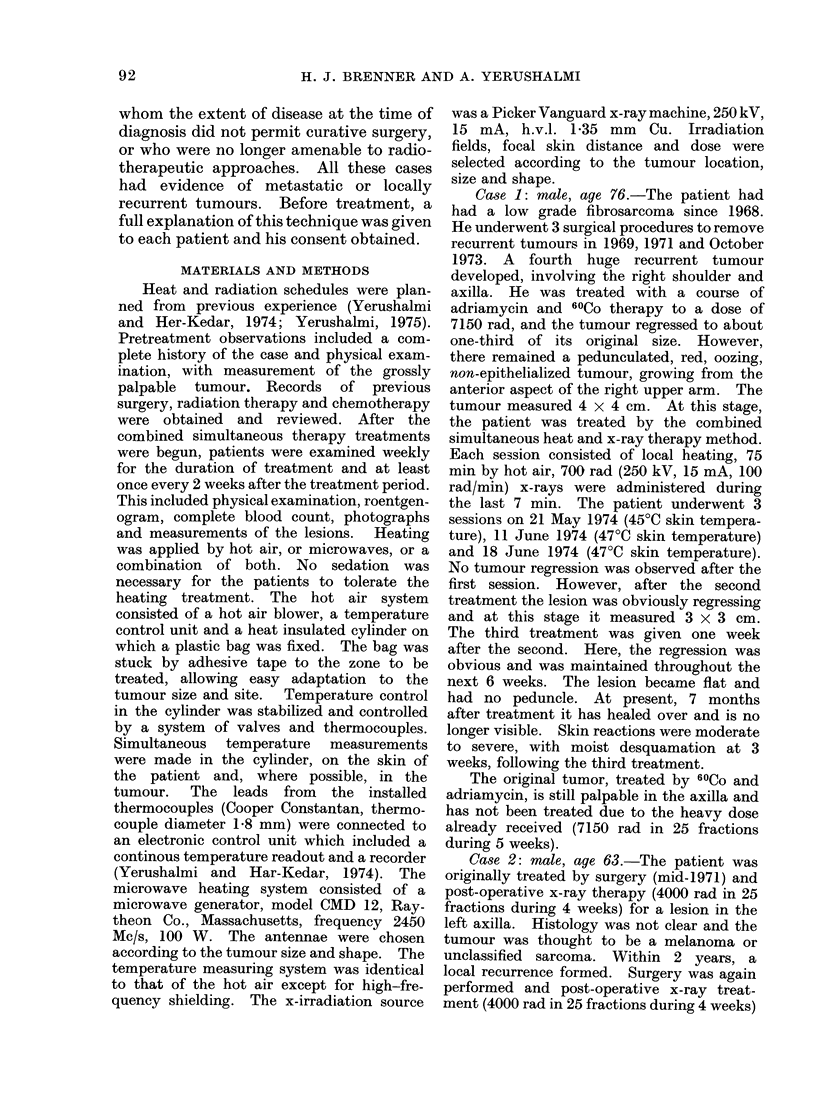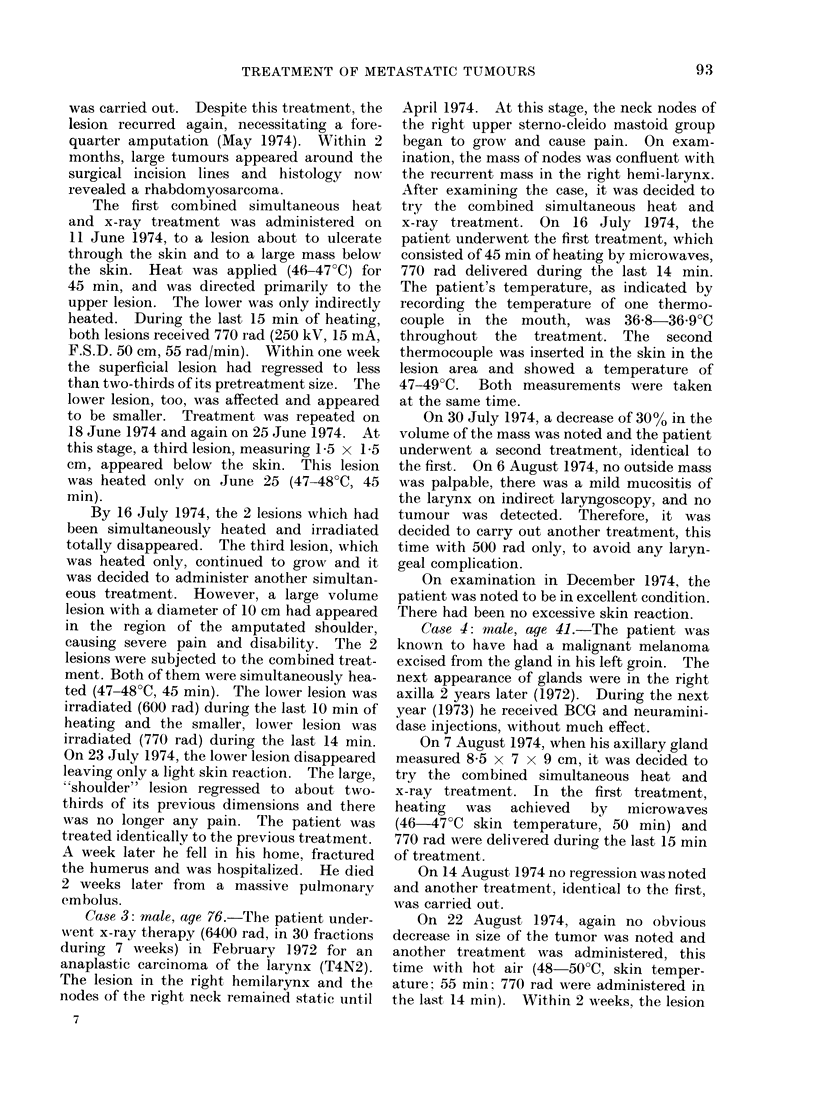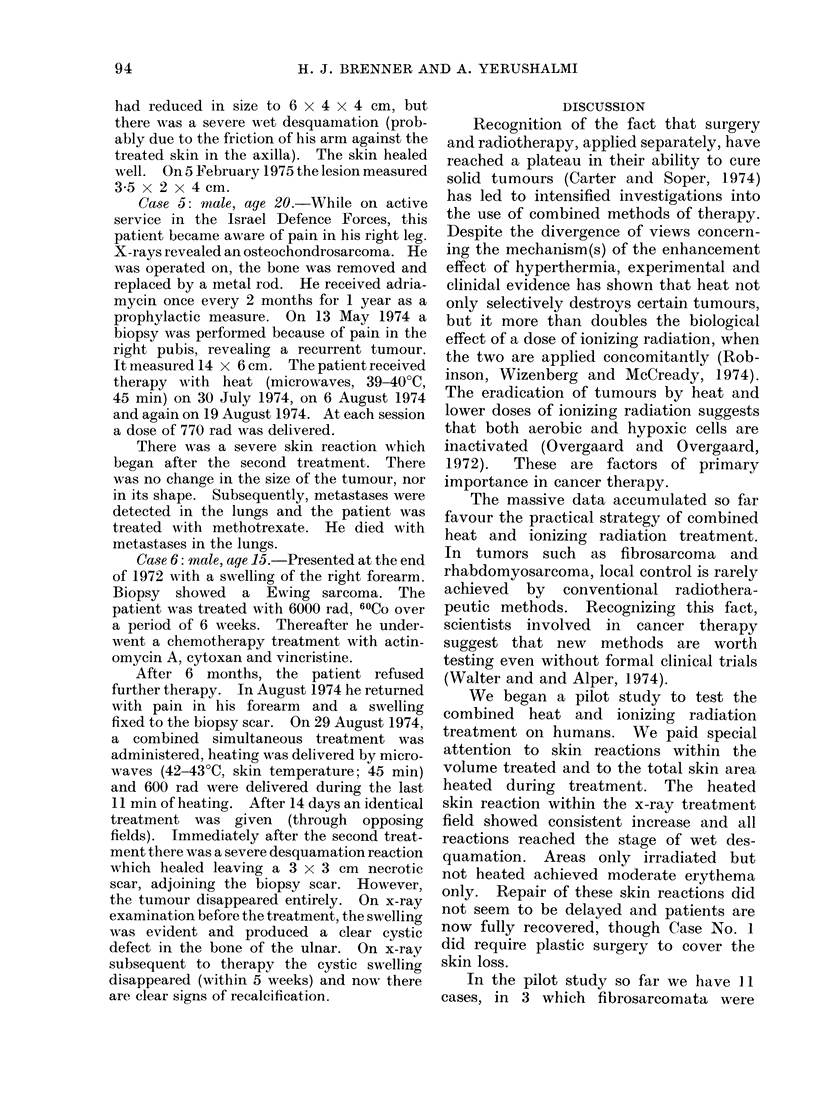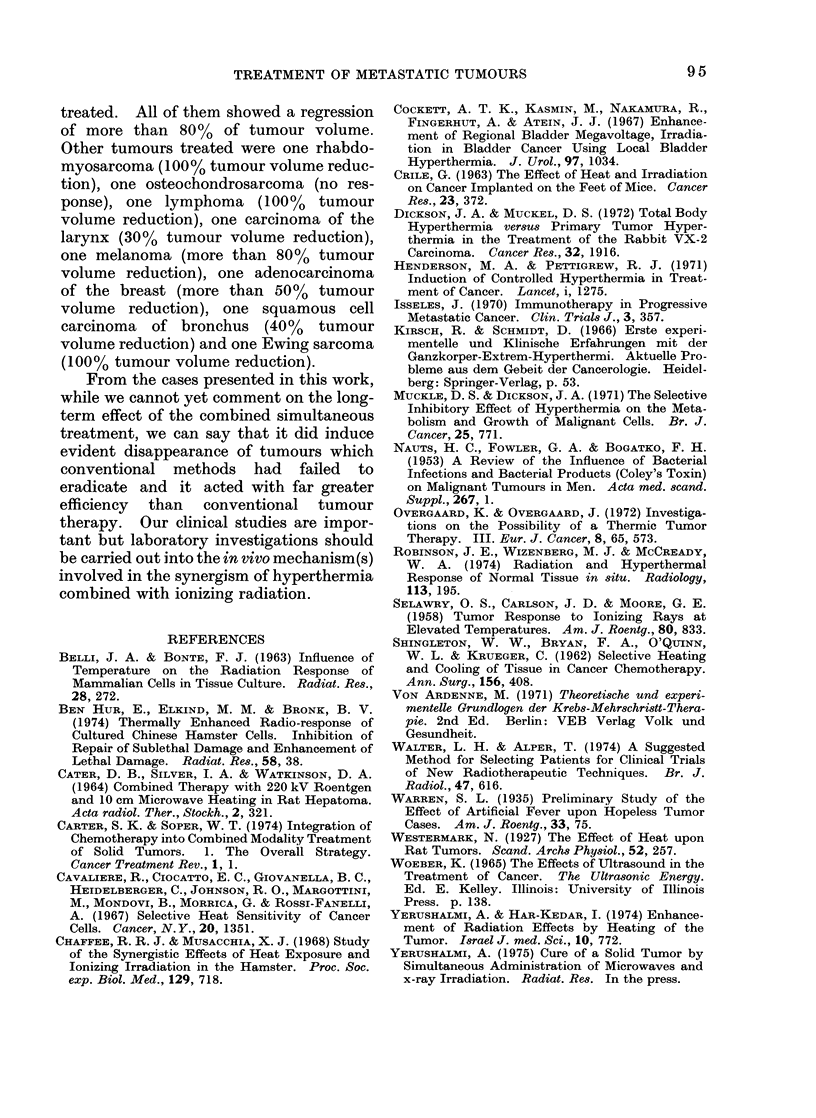# Combined local hyperthermia and x-irradiation in the treatment of metastatic tumours.

**DOI:** 10.1038/bjc.1976.9

**Published:** 1976-01

**Authors:** H. J. Brenner, A. Yerushalmi

## Abstract

Six patients, all with evidence of metastatic or locally recurrent tumours, were selected for inclusion in a trial study of simultaneous hyperthermia and ionizing radiation therapy. Heat was applied by hot air, or microwaves, or a combination of both. When examined after treatment, 3 patients were found to be free of the lesions treated. One patient had a partial response, followed by regression of the tumour; one patient died with metastases in the lungs and one patient responded to the treatment but died from a massive pulmonary embolus. The simultaneous application of hyperthermia and ionizing radiation therapy was well tolerated. It induced disappearance of tumours in cases where conventional methods had failed, and with far greater efficiency than conventional therapeutic methods.


*Br. J. Cancer* (1975) **33**, 91

### COMBINED LOCAL HYPERTHERMIA AND X-IRRADIATION IN THE TREATMENT OF METASTATIC TUMOURS

H. J. BRENNER* AND A. YERUSHALMI†

*From the *Department of Oncology, C. Sheba Medical Center, Tel Hashomer, Israel and †Radiation Unit, The Weizmann Institute of Science, Rehovot, Israel*

Received 13 August 1975 Accepted 19 September 1975

**Summary.—Six patients, all with evidence of metastatic or locally recurrent tumours, were selected for inclusion in a trial study of simultaneous hyperthermia and ionizing radiation therapy. Heat was applied by hot air, or microwaves, or a combination of both. When examined after treatment, 3 patients were found to be free of the lesions treated. One patient had a partial response, followed by regression of the tumour; one patient died with metastases in the lungs and one patient responded to the treatment but died from a massive pulmonary embolus. The simultaneous application of hyperthermia and ionizing radiation therapy was well tolerated. It induced disappearance of tumours in cases where conventional methods had failed, and with far greater efficiency than conventional therapeutic methods.**

HEAT has a selective inhibitory effect on cancer cells. Regression and destruction of established tumours in experimental animals and in humans has been achieved by pyrogens, by hyperthermia accompanying infectious diseases or by external physical heating (Cavaliere *et al.*, 1967; Isseles, 1970; Muckle and Dickson, 1971; Nauts, Fowler and Bogatko, 1953; Overgaard and Overgaard, 1972; Selawry, Carlson and Moore, 1958; Warren, 1935; Westermark, 1927). Various heating techniques have been tried, including total body hyperthermia by immersion in hot water (Kirsch and Schmidt, 1966; Von Ardenne, 1971) and by hot air inhalation (Henderson and Pettigrew, 1971), and local hyperthermia by hot water (Crile, 1963; Dickson and Muckel 1962), regional perfusion with heated fluids (Cavaliere *et al.*, 1967; Cockett *et al.*, 1967), short-wave diathermy (Overgaard and Overgaard, 1972; Shingleton *et al.*, 1962), ultrasound (Woeber, 1965), microwaves (Cater, Silver and Watkinson, 1964) and hot air (Yerushalmi and Har-Kedar, 1974). A system was developed by us (Yerushalmi and Har-Kedar, 1974) which allowed heating by hot air and concomitant irradiation of the tumour region. Using the simultaneous heat and radiation treatment, we obtained a cure of nearly 90% of tumour bearing mice. These studies provided clear evidence that heat produces a curative effect when applied simultaneously with radiation therapy. Earlier studies on mammals and mammalian cells had also shown that radiation sensitivity is modified by elevated temperatures (Belli and Bonte, 1963; Ben Hur, Elkind and Bronk, 1974; Chaffee and Musacchia, 1968). In clinical studies, patients with metastatic tumours had been treated by systemic hyperthermia (Warren, 1935) and cases with cancers in the limbs by regional perfusion with prewarmed blood to elevate the tumour temperature to the region of 42°C (Cavaliere *et al.*, 1967).

In the present work, we report on the treatment and results of the simultaneous heating and irradiation of patients in

This work was supported by a grant from the Gulton Foundation, Englewood, New Jersey, U.S.A.
Reprint requests to: A. Yerushalmi, Radiation Unit, The Weizmann Institute of Science, Rehovot, Israel.

whom the extent of disease at the time of diagnosis did not permit curative surgery, or who were no longer amenable to radiotherapeutic approaches. All these cases had evidence of metastatic or locally recurrent tumours. Before treatment, a full explanation of this technique was given to each patient and his consent obtained.

### MATERIALS AND METHODS

Heat and radiation schedules were planned from previous experience (Yerushalmi and Her-Kedar, 1974; Yerushalmi, 1975). Pretreatment observations included a complete history of the case and physical examination, with measurement of the grossly palpable tumour. Records of previous surgery, radiation therapy and chemotherapy were obtained and reviewed. After the combined simultaneous therapy treatments were begun, patients were examined weekly for the duration of treatment and at least once every 2 weeks after the treatment period. This included physical examination, roentgenogram, complete blood count, photographs and measurements of the lesions. Heating was applied by hot air, or microwaves, or a combination of both. No sedation was necessary for the patients to tolerate the heating treatment. The hot air system consisted of a hot air blower, a temperature control unit and a heat insulated cylinder on which a plastic bag was fixed. The bag was stuck by adhesive tape to the zone to be treated, allowing easy adaptation to the tumour size and site. Temperature control in the cylinder was stabilized and controlled by a system of valves and thermocouples. Simultaneous temperature measurements were made in the cylinder, on the skin of the patient and, where possible, in the tumour. The leads from the installed thermocouples (Cooper Constantan, thermocouple diameter 1·8 mm) were connected to an electronic control unit which included a continous temperature readout and a recorder (Yerushalmi and Har-Kedar, 1974). The microwave heating system consisted of a microwave generator, model CMD 12, Raytheon Co., Massachusetts, frequency 2450 Mc/s, 100 W. The antennae were chosen according to the tumour size and shape. The temperature measuring system was identical to that of the hot air except for high–frequency shielding. The x-irradiation source was a Picker Vanguard x-ray machine, 250 kV, 15 mA, h.v.l. 1·35 mm Cu. Irradiation fields, focal skin distance and dose were selected according to the tumour location, size and shape.

*Case 1*: *male, age 76*.—The patient had had a low grade fibrosarcoma since 1968. He underwent 3 surgical procedures to remove recurrent tumours in 1969, 1971 and October 1973. A fourth huge recurrent tumour developed, involving the right shoulder and axilla. He was treated with a course of adriamycin and $^{60}Co$ therapy to a dose of 7150 rad, and the tumour regressed to about one-third of its original size. However, there remained a pedunculated, red, oozing, *non*-epithelialized tumour, growing from the anterior aspect of the right upper arm. The tumour measured 4 × 4 cm. At this stage, the patient was treated by the combined simultaneous heat and x-ray therapy method. Each session consisted of local heating, 75 min by hot air, 700 rad (250 kV, 15 mA, 100 rad/min) x-rays were administered during the last 7 min. The patient underwent 3 sessions on 21 May 1974 (45°C skin temperature), 11 June 1974 (47°C skin temperature) and 18 June 1974 (47°C skin temperature). No tumour regression was observed after the first session. However, after the second treatment the lesion was obviously regressing and at this stage it measured 3 × 3 cm. The third treatment was given one week after the second. Here, the regression was obvious and was maintained throughout the next 6 weeks. The lesion became flat and had no peduncle. At present, 7 months after treatment it has healed over and is no longer visible. Skin reactions were moderate to severe, with moist desquamation at 3 weeks, following the third treatment.

The original tumor, treated by $^{60}Co$ and adriamycin, is still palpable in the axilla and has not been treated due to the heavy dose already received (7150 rad in 25 fractions during 5 weeks).

*Case 2*: *male, age 63*.—The patient was originally treated by surgery (mid-1971) and post-operative x-ray therapy (4000 rad in 25 fractions during 4 weeks) for a lesion in the left axilla. Histology was not clear and the tumour was thought to be a melanoma or unclassified sarcoma. Within 2 years, a local recurrence formed. Surgery was again performed and post-operative x-ray treatment (4000 rad in 25 fractions during 4 weeks)

was carried out. Despite this treatment, the lesion recurred again, necessitating a forequarter amputation (May 1974). Within 2 months, large tumours appeared around the surgical incision lines and histology now revealed a rhabdomyosarcoma.

The first combined simultaneous heat and x-ray treatment was administered on 11 June 1974, to a lesion about to ulcerate through the skin and to a large mass below the skin. Heat was applied (46–47°C) for 45 min, and was directed primarily to the upper lesion. The lower was only indirectly heated. During the last 15 min of heating, both lesions received 770 rad (250 kV, 15 mA, F.S.D. 50 cm, 55 rad/min). Within one week the superficial lesion had regressed to less than two-thirds of its pretreatment size. The lower lesion, too, was affected and appeared to be smaller. Treatment was repeated on 18 June 1974 and again on 25 June 1974. At this stage, a third lesion, measuring 1·5 × 1·5 cm, appeared below the skin. This lesion was heated only on June 25 (47–48°C, 45 min).

By 16 July 1974, the 2 lesions which had been simultaneously heated and irradiated totally disappeared. The third lesion, which was heated only, continued to grow and it was decided to administer another simultaneous treatment. However, a large volume lesion with a diameter of 10 cm had appeared in the region of the amputated shoulder, causing severe pain and disability. The 2 lesions were subjected to the combined treatment. Both of them were simultaneously heated (47–48°C, 45 min). The lower lesion was irradiated (600 rad) during the last 10 min of heating and the smaller, lower lesion was irradiated (770 rad) during the last 14 min. On 23 July 1974, the lower lesion disappeared leaving only a light skin reaction. The large, "shoulder" lesion regressed to about two-thirds of its previous dimensions and there was no longer any pain. The patient was treated identically to the previous treatment. A week later he fell in his home, fractured the humerus and was hospitalized. He died 2 weeks later from a massive pulmonary embolus.

*Case 3*: *male, age 76.*—The patient underwent x-ray therapy (6400 rad, in 30 fractions during 7 weeks) in February 1972 for an anaplastic carcinoma of the larynx (T4N2). The lesion in the right hemilarynx and the nodes of the right neck remained static until April 1974. At this stage, the neck nodes of the right upper sterno-cleido mastoid group began to grow and cause pain. On examination, the mass of nodes was confluent with the recurrent mass in the right hemi-larynx. After examining the case, it was decided to try the combined simultaneous heat and x-ray treatment. On 16 July 1974, the patient underwent the first treatment, which consisted of 45 min of heating by microwaves, 770 rad delivered during the last 14 min. The patient's temperature, as indicated by recording the temperature of one thermocouple in the mouth, was 36·8—36·9°C throughout the treatment. The second thermocouple was inserted in the skin in the lesion area and showed a temperature of 47–49°C. Both measurements were taken at the same time.

On 30 July 1974, a decrease of 30% in the volume of the mass was noted and the patient underwent a second treatment, identical to the first. On 6 August 1974, no outside mass was palpable, there was a mild mucositis of the larynx on indirect laryngoscopy, and no tumour was detected. Therefore, it was decided to carry out another treatment, this time with 500 rad only, to avoid any laryngeal complication.

On examination in December 1974, the patient was noted to be in excellent condition. There had been no excessive skin reaction.

*Case 4*: *male, age 41.*—The patient was known to have had a malignant melanoma excised from the gland in his left groin. The next appearance of glands were in the right axilla 2 years later (1972). During the next year (1973) he received BCG and neuraminidase injections, without much effect.

On 7 August 1974, when his axillary gland measured 8·5 × 7 × 9 cm, it was decided to try the combined simultaneous heat and x-ray treatment. In the first treatment, heating was achieved by microwaves (46—47°C skin temperature, 50 min) and 770 rad were delivered during the last 15 min of treatment.

On 14 August 1974 no regression was noted and another treatment, identical to the first, was carried out.

On 22 August 1974, again no obvious decrease in size of the tumor was noted and another treatment was administered, this time with hot air (48—50°C, skin temperature; 55 min; 770 rad were administered in the last 14 min). Within 2 weeks, the lesion had reduced in size to 6 × 4 × 4 cm, but there was a severe wet desquamation (probably due to the friction of his arm against the treated skin in the axilla). The skin healed well. On 5 February 1975 the lesion measured 3·5 × 2 × 4 cm.

*Case 5: male, age 20.*—While on active service in the Israel Defence Forces, this patient became aware of pain in his right leg. X-rays revealed an osteochondrosarcoma. He was operated on, the bone was removed and replaced by a metal rod. He received adriamycin once every 2 months for 1 year as a prophylactic measure. On 13 May 1974 a biopsy was performed because of pain in the right pubis, revealing a recurrent tumour. It measured 14 × 6 cm. The patient received therapy with heat (microwaves, 39–40°C, 45 min) on 30 July 1974, on 6 August 1974 and again on 19 August 1974. At each session a dose of 770 rad was delivered.

There was a severe skin reaction which began after the second treatment. There was no change in the size of the tumour, nor in its shape. Subsequently, metastases were detected in the lungs and the patient was treated with methotrexate. He died with metastases in the lungs.

*Case 6: male, age 15.*—Presented at the end of 1972 with a swelling of the right forearm. Biopsy showed a Ewing sarcoma. The patient was treated with 6000 rad, $^{60}Co$ over a period of 6 weeks. Thereafter he underwent a chemotherapy treatment with actinomycin A, cytoxan and vincristine.

After 6 months, the patient refused further therapy. In August 1974 he returned with pain in his forearm and a swelling fixed to the biopsy scar. On 29 August 1974, a combined simultaneous treatment was administered, heating was delivered by microwaves (42–43°C, skin temperature; 45 min) and 600 rad were delivered during the last 11 min of heating. After 14 days an identical treatment was given (through opposing fields). Immediately after the second treatment there was a severe desquamation reaction which healed leaving a 3 × 3 cm necrotic scar, adjoining the biopsy scar. However, the tumour disappeared entirely. On x-ray examination before the treatment, the swelling was evident and produced a clear cystic defect in the bone of the ulnar. On x-ray subsequent to therapy the cystic swelling disappeared (within 5 weeks) and now there are clear signs of recalcification.

### DISCUSSION

Recognition of the fact that surgery and radiotherapy, applied separately, have reached a plateau in their ability to cure solid tumours (Carter and Soper, 1974) has led to intensified investigations into the use of combined methods of therapy. Despite the divergence of views concerning the mechanism(s) of the enhancement effect of hyperthermia, experimental and clinidal evidence has shown that heat not only selectively destroys certain tumours, but it more than doubles the biological effect of a dose of ionizing radiation, when the two are applied concomitantly (Robinson, Wizenberg and McCready, 1974). The eradication of tumours by heat and lower doses of ionizing radiation suggests that both aerobic and hypoxic cells are inactivated (Overgaard and Overgaard, 1972). These are factors of primary importance in cancer therapy.

The massive data accumulated so far favour the practical strategy of combined heat and ionizing radiation treatment. In tumors such as fibrosarcoma and rhabdomyosarcoma, local control is rarely achieved by conventional radiotherapeutic methods. Recognizing this fact, scientists involved in cancer therapy suggest that new methods are worth testing even without formal clinical trials (Walter and and Alper, 1974).

We began a pilot study to test the combined heat and ionizing radiation treatment on humans. We paid special attention to skin reactions within the volume treated and to the total skin area heated during treatment. The heated skin reaction within the x-ray treatment field showed consistent increase and all reactions reached the stage of wet desquamation. Areas only irradiated but not heated achieved moderate erythema only. Repair of these skin reactions did not seem to be delayed and patients are now fully recovered, though Case No. 1 did require plastic surgery to cover the skin loss.

In the pilot study so far we have 11 cases, in 3 which fibrosarcomata were treated. All of them showed a regression of more than 80% of tumour volume. Other tumours treated were one rhabdomyosarcoma (100% tumour volume reduction), one osteochondrosarcoma (no response), one lymphoma (100% tumour volume reduction), one carcinoma of the larynx (30% tumour volume reduction), one melanoma (more than 80% tumour volume reduction), one adenocarcinoma of the breast (more than 50% tumour volume reduction), one squamous cell carcinoma of bronchus (40% tumour volume reduction) and one Ewing sarcoma (100% tumour volume reduction).

From the cases presented in this work, while we cannot yet comment on the long-term effect of the combined simultaneous treatment, we can say that it did induce evident disappearance of tumours which conventional methods had failed to eradicate and it acted with far greater efficiency than conventional tumour therapy. Our clinical studies are important but laboratory investigations should be carried out into the *in vivo* mechanism(s) involved in the synergism of hyperthermia combined with ionizing radiation.

### REFERENCES

Belli, J. A. & Bonte, F. J. (1963) Influence of Temperature on the Radiation Response of Mammalian Cells in Tissue Culture. *Radiat. Res.*, **28**, 272.

Ben Hur, E., Elkind, M. M. & Bronk, B. V. (1974) Thermally Enhanced Radio-response of Cultured Chinese Hamster Cells. Inhibition of Repair of Sublethal Damage and Enhancement of Lethal Damage. *Radiat. Res.*, **58**, 38.

Cater, D. B., Silver, I. A. & Watkinson, D. A. (1964) Combined Therapy with 220 kV Roentgen and 10 cm Microwave Heating in Rat Hepatoma. *Acta radiol. Ther., Stockh.*, **2**, 321.

Carter, S. K. & Soper, W. T. (1974) Integration of Chemotherapy into Combined Modality Treatment of Solid Tumors. 1. The Overall Strategy. *Cancer Treatment Rev.*, **1**, 1.

Cavaliere, R., Ciocatto, E. C., Giovanella, B. C., Heidelberger, C., Johnson, R. O., Margottini, M., Mondovi, B., Morrica, G. & Rossi-Fanelli, A. (1967) Selective Heat Sensitivity of Cancer Cells. *Cancer, N.Y.*, **20**, 1351.

Chaffee, R. R. J. & Musacchia, X. J. (1968) Study of the Synergistic Effects of Heat Exposure and Ionizing Irradiation in the Hamster. *Proc. Soc. exp. Biol. Med.*, **129**, 718.

Cockett, A. T. K., Kasmin, M., Nakamura, R., Fingerhut, A. & Atein, J. J. (1967) Enhancement of Regional Bladder Megavoltage, Irradiation in Bladder Cancer Using Local Bladder Hyperthermia. *J. Urol.*, **97**, 1034.

Crile, G. (1963) The Effect of Heat and Irradiation on Cancer Implanted on the Feet of Mice. *Cancer Res.*, **23**, 372.

Dickson, J. A. & Muckel, D. S. (1972) Total Body Hyperthermia *versus* Primary Tumor Hyperthermia in the Treatment of the Rabbit VX-2 Carcinoma. *Cancer Res.*, **32**, 1916.

Henderson, M. A. & Pettigrew, R. J. (1971) Induction of Controlled Hyperthermia in Treatment of Cancer. *Lancet*, i, 1275.

Isseles, J. (1970) Immunotherapy in Progressive Metastatic Cancer. *Clin. Trials J.*, **3**, 357.

Kirsch, R. & Schmidt, D. (1966) Erste experimentelle und Klinische Erfahrungen mit der Ganzkorper-Extrem-Hyperthermi. Aktuelle Probleme aus dem Gebeit der Cancerologie. Heidelberg: Springer-Verlag, p. 53.

Muckle, D. S. & Dickson, J. A. (1971) The Selective Inhibitory Effect of Hyperthermia on the Metabolism and Growth of Malignant Cells. *Br. J. Cancer*, **25**, 771.

Nauts, H. C., Fowler, G. A. & Bogatko, F. H. (1953) A Review of the Influence of Bacterial Infections and Bacterial Products (Coley's Toxin) on Malignant Tumours in Men. *Acta med. scand. Suppl.*, **267**, 1.

Overgaard, K. & Overgaard, J. (1972) Investigations on the Possibility of a Thermic Tumor Therapy. III. *Eur. J. Cancer*, **8**, 65, 573.

Robinson, J. E., Wizenberg, M. J. & McCready, W. A. (1974) Radiation and Hyperthermal Response of Normal Tissue *in situ*. *Radiology*, **113**, 195.

Selawry, O. S., Carlson, J. D. & Moore, G. E. (1958) Tumor Response to Ionizing Rays at Elevated Temperatures. *Am. J. Roentg.*, **80**, 833.

Shingleton, W. W., Bryan, F. A., O'Quinn, W. L. & Krueger, C. (1962) Selective Heating and Cooling of Tissue in Cancer Chemotherapy. *Ann. Surg.*, **156**, 408.

Von Ardenne, M. (1971) *Theoretische und experimentelle Grundlogen der Krebs-Mehrschristt-Therapie.* 2nd Ed. Berlin: VEB Verlag Volk und Gesundheit.

Walter, L. H. & Alper, T. (1974) A Suggested Method for Selecting Patients for Clinical Trials of New Radiotherapeutic Techniques. *Br. J. Radiol.*, **47**, 616.

Warren, S. L. (1935) Preliminary Study of the Effect of Artificial Fever upon Hopeless Tumor Cases. *Am. J. Roentg.*, **33**, 75.

Westermark, N. (1927) The Effect of Heat upon Rat Tumors. *Scand. Archs Physiol.*, **52**, 257.

Woeber, K. (1965) The Effects of Ultrasound in the Treatment of Cancer. *The Ultrasonic Energy.* Ed. E. Kelley. Illinois: University of Illinois Press. p. 138.

Yerushalmi, A. & Har-Kedar, I. (1974) Enhancement of Radiation Effects by Heating of the Tumor. *Israel J. med. Sci.*, **10**, 772.

Yerushalmi, A. (1975) Cure of a Solid Tumor by Simultaneous Administration of Microwaves and x-ray Irradiation. *Radiat. Res.* In the press.